# Supplementary material for: Analyzing Hierarchical Multi-View MRI Data With StaPLR: An Application to Alzheimer's Disease Classification
Source: Front Neurosci. 2022 Apr 25;16:830630. doi: 10.3389/fnins.2022.830630 (PMC9082949; doi:10.3389/fnins.2022.830630)
Supplement: Supplementary file 1 [file Data_Sheet_1.PDF]

Supplementary Material

1 INTRODUCTION

This document describes the process of obtaining the features used in “Analyzing hierarchical multi-view MRI data with StaPLR: An application to Alzheimer’s disease classification”. This document is largely a reiteration of previous work, in particular that of de Vos et al. (2016, 2017) and Schouten et al. (2016, 2017). The relevant information from these publications is collected here for the reader’s convenience.

2 PARTICIPANTS

The data were collected at the Medical University of Graz in Austria, and consisted of 76 clinically diagnosed probable AD patients and 173 cognitively normal elderly. The AD patients were part of the prospective registry on dementia (PRODEM) (Seiler et al., 2012). The inclusion criteria for PRODEM are: Dementia diagnosis according to DSM-IV criteria (DSM, 2000), AD diagnosis according to the NINCDS-ADRDA criteria (McKhann et al., 2011), non-institutionalisation or need for 24-h care, and the availability of a caregiver who agrees to provide information on the patients’ and his or her own condition. Patients were excluded if co-morbidities were likely to preclude successful completion of the study. Informed consent was obtained from all patients and their caregivers. We only included patients for which anatomical MRI, diffusion MRI and rs-fMRI were available. The controls were scanned at the same scanning site, over the same period, with the same scanning protocol as the AD patients as a part of the Austrian Stroke Prevention Study (ASPS). The ASPS is a community-based cohort study on the effects of vascular risk factors on brain structure and function in elderly participants without a history or signs of stroke and dementia on the inhabitants of Graz, Austria (Schmidt et al., 1994; Freudenberger et al., 2016). Informed consent was obtained from all participants.

3 MRI ACQUISITION

Each participant was scanned on a Siemens Magnetom TrioTim 3 T MRI scanner. Anatomical T1-weighted images were acquired with TR = 1900 ms, TE = 2.19 ms, flip angle = 9, 179 slices, with an isotropic voxel size of 1 mm.

Diffusion images were acquired along 12 non-collinear directions, scanning each direction 4 times with TR = 6700 ms, TE = 95 ms, 50 axial slices, voxel size = $2.0 \times 2.0 \times 2.5$ mm.

Resting-state fMRI series of 150 volumes were obtained with TR = 3000 ms, TE = 30 ms, flip angle = 90, 40 axial slices, with an isotropic voxel size of 3 mm. Participants were instructed to lie still with their eyes closed, and to stay awake.

4 MRI PREPROCESSING

The MRI data of all subjects were preprocessed using the FMRIB Software Library (FSL version 5.0) (Jenkinson et al., 2012; Smith et al., 2004). For the anatomical MRI scans, we applied brain extraction and bias field correction. For the diffusion MRI scans, we applied brain extraction and eddy current correction. For the rs-fMRI data, this included brain extraction, motion correction, a temporal high pass filter with a cutoff point of 100 seconds, 3 mm FWHM spatial smoothing, and non-linear registration to standard MNI152 space. Additionally, we used ICA-AROMA to automatically identify and remove noise components from the fMRI time course (Pruim et al., 2015). ICA-AROMA adequately removes motion

related noise from fMRI data, without the need for removing volumes with excessive motion (Parkes et al., 2018).

5 FEATURE EXTRACTION

5.1 Structural MRI

The process of extracting the features corresponding to cortical thickness, area, curvature, grey matter density, and subcortical volumes, is identical to the process described in de Vos et al. (2016). For completeness, we also describe it below.

In order to calculate cortical thickness, cortical area and cortical curvature, the raw (not preprocessed) T1-weighted images were processed using Freesurfer 5.3.0 (Dale et al., 1999; Fischl et al., 1999). First, this entails intensity normalization and brain extraction (de Vos et al., 2016). Using the resulting image the boundary between grey and white matter was located, and a triangular mesh was constructed around the white matter surface (de Vos et al., 2016). The grey matter surface was created by deforming the mesh outward so that it closely followed the boundary between grey matter and cerebral spinal fluid (de Vos et al., 2016). Cortical thickness was calculated as the distance between the white matter and grey matter surface for each vertex (de Vos et al., 2016). The image was then registered to the Freesurfer common template using the image's cortical folding pattern (de Vos et al., 2016). The neocortex was parcellated into the 68 regions of the Desikan-Killiany atlas (Desikan et al., 2006). The thickness of each parcellation unit was calculated as the mean thickness of all the vertices within that parcellation (de Vos et al., 2016). Thus, 68 cortical thickness features are obtained per subject. The cortical surface area was calculated by summing the areas of the grey matter mesh triangles for each parcellation, yielding 68 cortical area features per subject (de Vos et al., 2016). To obtain the cortical curvature features, the mean of the curvature values in the two principal directions of the of the surface was calculated (de Vos et al., 2016). The curvature of a vertex in these directions was calculated as the inverse of the length of the radius of osculating circles in these directions (Ronan et al., 2011). The curvature values of the vertices were averaged for each of the parcellations, yielding 68 cortical curvature features per subject (de Vos et al., 2016).

Grey matter density was calculated using FSL VBM (version 5.0.7) (Ashburner and Friston, 2000; Smith et al., 2004). The brain-extracted images were first segmented into grey matter, white matter, and CSF (de Vos et al., 2016). A study-specific grey matter template was created in two steps. First, the grey matter images were affine-registered to the ICBM-152 grey matter template and the resulting images were averaged to create a first-pass template (de Vos et al., 2016). Then, the grey matter images were nonlinearly registered to the first-pass temlate and the resulting images were averaged to obtain a final template at $2 \times 2 \times 2\text{mm}^3$ resolution in standard space (de Vos et al., 2016). The grey matter images were then registered to the final template and smoothed with a Gaussian kernel with a full width at half maximum of 3 mm (de Vos et al., 2016). The voxel wise values were then averaged within the 48 regions of the probabilistic Harvard-Oxford cortical atlas (de Vos et al., 2016). The 48 grey matter density features were obtained by calculating the weighted averages of the regions, with voxels contribution to the average of a region based on their probability of being part of that region (de Vos et al., 2016).

The volumes of the subcortical structures were calculated using the FMRIB's Integrated Registration and Segmentation Tool (FIRST) in FSL (Patenaude et al., 2011). The whole-head images were affine registered to the nonlinear MNI-152 template (de Vos et al., 2016). In a second stage, initialized by the result of the first stage, a subcortical mask was used to achieve a more accurate and robust affine registration (de Vos et al., 2016). The shapes of the subcortical structures were modeled by deformable meshes and the boundary voxels were classified as being part of the subcortical structure using structural segmentation

(Zhang et al., 2001). The cortical volumes were then corrected for intracranial volume as obtained by FSL, yielding 14 subcortical volume features per subject, corresponding to the thalamus, caudate, putamen, pallidum, hippocampus, amygdala and accumbens of both hemispheres (de Vos et al., 2016).

5.2 Diffusion-weighted MRI

The diffusion MRI scans were used to calculate fractional anisotropy (FA), mean diffusivity (MD), axial diffusivity (DA), and radial diffusivity (DR). First, DTIFIT in FSL (Jenkinson et al., 2012; Smith et al., 2004) was used to fit a diffusion tensor model at each voxel to calculate voxel-wise FA, MD, DA and DR images for each subject. Then subjects' FA, MD, DA and DR images were projected onto the FMRIB58_FA mean FA image using tract based spatial statistics (TBSS) (Smith et al., 2006). Finally, weighted averages of the FA, MD, DA and DR values were calculated within the 20 regions of the probabilistic JHU white-matter tractography atlas (Hua et al., 2008), yielding 20 features for FA as well as MD, DA and DR (Schouten et al., 2016).

5.3 Resting state fMRI

The resting state fMRI feature sets used in this article have already been described in detail in de Vos et al. (2017); the following is a reiteration of the most relevant sections.

Resting state networks (RSNs) were obtained using temporal concatenation independent component analysis (ICA) in FSL MELODIC (Beckmann and Smith, 2004). The functional data of all participants was registered to standard space and concatenated along the time dimension. ICA was performed on the concatenated dataset, once with 20 and once with 70 components (de Vos et al., 2017). The resulting ICA component weight maps were registered back to subject space, weighted by subject specific grey matter density maps, and multiplied with the functional data, resulting in mean time course for each component (de Vos et al., 2017). These time course were then used to calculate functional connectivity matrices using both full and sparse partial correlations (de Vos et al., 2017). The partial correlation matrices were calculated using the graphical lasso (Friedman et al., 2008) implemented in MATLAB (Inc., 2013), with $\lambda = 100$ (de Vos et al., 2017). The resulting two 20×20 matrices contain 190 unique elements, and the 70×70 matrices contain 2415 unique elements to be used as candidate features in the classification. Any features with zero variance were removed.

The dynamics of the FC matrices were calculated using a sliding window approach with a window size of 33s (de Vos et al., 2017). The windows were shifted one volume at a time, leading to 140 windows (de Vos et al., 2017). The previously described four FC matrices were calculated within each window, and the standard deviation of the FC matrices of all windows was obtained (de Vos et al., 2017).

The sliding window FC matrices were clustered using k-means clustering (with k=5 and Manhattan distance) to obtain 5 ”FC states” (de Vos et al., 2017). The number of sliding window matrices that were assigned to each of the five FC states was then calculated for each participant (de Vos et al., 2017).

Graph metrics were calculated using the Brain Connectivity Toolbox (Rubinov and Sporns, 2010) in MATLAB (Inc., 2013) for each of the four FC matrices. Connection strength, weighted betweenness centrality and weighted clustering coefficients were calculated for every node, and weighted characteristic path length and weighted transitivity for the entire network (de Vos et al., 2017). Additionally, several graph metrics were calculated on binarized versions of the FC matrices: connection degree, betweenness centrality and clustering coefficient for every node, and characteristic path length and transitivity for the entire network (de Vos et al., 2017)

Whole brain FC with 10 RSNs was calculated using dual regression in FSL (Filippini et al., 2009), using the RSN templates of Smith et al. (2012). The voxel-wise whole brain FC results for each of the 10 RSNs

were used as 10 distinct feature sets. Voxel-wise whole brain FC maps were additionally obtained for the left and right hippocampus (de Vos et al., 2017).

Eigenvector centrality maps were calculated for each participant using fastECM (Wink et al., 2012; Binnewijzend et al., 2014)

The amplitude of low frequency fluctuations (ALFF) (Yu-Feng et al., 2007; Biswal et al., 2010) and fractional ALFF (Zou et al., 2008) were calculated for each participant using REST (Song et al., 2011). The voxels' ALFF and fALFF values were divided by the mean ALFF/fALFF within a subjects whole brain (Zou et al., 2008; de Vos et al., 2017).

REFERENCES

(2000). *Diagnostic and statistical manual of mental disorders* (American Psychiatric Association, Washington, DC), 4th edn.

Ashburner, J. and Friston, K. J. (2000). Voxel-based morphometry—the methods. *NeuroImage* 11, 805–821

Beckmann, C. F. and Smith, S. M. (2004). Probabilistic independent component analysis for functional magnetic resonance imaging. *IEEE transactions on medical imaging* 23, 137–152

Binnewijzend, M. A., Adriaanse, S. M., Van der Flier, W. M., Teunissen, C. E., de Munck, J. C., Stam, C. J., et al. (2014). Brain network alterations in alzheimer's disease measured by eigenvector centrality in fmri are related to cognition and csf biomarkers. *Human brain mapping* 35, 2383–2393

Biswal, B. B., Mennes, M., Zuo, X.-N., Gohel, S., Kelly, C., Smith, S. M., et al. (2010). Toward discovery science of human brain function. *Proceedings of the National Academy of Sciences* 107, 4734–4739

Dale, A. M., Fischl, B., and Sereno, M. I. (1999). Cortical surface-based analysis: I. segmentation and surface reconstruction. *NeuroImage* 9, 179–194

de Vos, F., Koini, M., Schouten, T., Seiler, S., van der Grond, J., Lechner, A., et al. (2017). A comprehensive analysis of resting state fMRI measures to classify individual patients with Alzheimer's disease. *NeuroImage* 167, 62–72

de Vos, F., Schouten, T., Hafkemeijer, A., Dopper, E., van Swieten, J., de Rooij, M., et al. (2016). Combining multiple anatomical MRI measures improves Alzheimer's disease classification. *Human Brain Mapping* 37, 1920–1929

Desikan, R. S., Ségonne, F., Fischl, B., Quinn, B. T., Dickerson, B. C., Blacker, D., et al. (2006). An automated labeling system for subdividing the human cerebral cortex on MRI scans into gyral based regions of interest. *NeuroImage* 31, 968–980

Filippini, N., MacIntosh, B., Hough, M., Goodwin, G., Frisoni, G., Ebmeier, K., et al. (2009). Distinct patterns of brain activity in young carriers of the apoe e4 allele. *NeuroImage* 47, S139–S139

Fischl, B., Sereno, M. I., and Dale, A. M. (1999). Cortical surface-based analysis: Ii: inflation, flattening, and a surface-based coordinate system. *NeuroImage* 9, 195–207

Freudenberger, P., Petrovic, K., Sen, A., Töglhofer, A. M., Fixa, A., Hofer, E., et al. (2016). Fitness and cognition in the elderly: the Austrian stroke prevention study. *Neurology* 86, 418–424

Friedman, J., Hastie, T., and Tibshirani, R. (2008). Sparse inverse covariance estimation with the graphical lasso. *Biostatistics* 9, 432–441

Hua, K., Zhang, J., Wakana, S., Jiang, H., Li, X., Reich, D. S., et al. (2008). Tract probability maps in stereotaxic spaces: analyses of white matter anatomy and tract-specific quantification. *NeuroImage* 39, 336–347

Inc., T. M. (2013). *MATLAB and Statistics Toolbox Release, 2013a*

Jenkinson, M., Beckmann, C. F., Behrens, T. E., Woolrich, M. W., and Smith, S. M. (2012). Fsl. *NeuroImage* 62, 782–790

McKhann, G. M., Knopman, D. S., Chertkow, H., Hyman, B. T., Jack Jr, C. R., Kawas, C. H., et al. (2011). The diagnosis of dementia due to alzheimer's disease: recommendations from the national institute on aging-alzheimer's association workgroups on diagnostic guidelines for alzheimer's disease. *Alzheimer's & dementia* 7, 263–269

Parkes, L., Fulcher, B., Yücel, M., and Fornito, A. (2018). An evaluation of the efficacy, reliability, and sensitivity of motion correction strategies for resting-state functional mri. *NeuroImage* 171, 415–436

Patenaude, B., Smith, S. M., Kennedy, D. N., and Jenkinson, M. (2011). A bayesian model of shape and appearance for subcortical brain segmentation. *NeuroImage* 56, 907–922

Pruim, R. H., Mennes, M., van Rooij, D., Llera, A., Buitelaar, J. K., and Beckmann, C. F. (2015). Ica-aroma: A robust ica-based strategy for removing motion artifacts from fmri data. *NeuroImage* 112, 267–277

Ronan, L., Pienaar, R., Williams, G., Bullmore, E., Crow, T. J., Roberts, N., et al. (2011). Intrinsic curvature: a marker of millimeter-scale tangential cortico-cortical connectivity? *International journal of neural systems* 21, 351–366

Rubinov, M. and Sporns, O. (2010). Complex network measures of brain connectivity: uses and interpretations. *NeuroImage* 52, 1059–1069

Schmidt, R., Lechner, H., Fazekas, F., Niederkorn, K., Reinhart, B., Grieshofer, P., et al. (1994). Assessment of cerebrovascular risk profiles in healthy persons: definition of research goals and the Austrian stroke prevention study (ASPS). *Neuroepidemiology* 13, 308–313

Schouten, T., Koini, M., De Vos, F., Seiler, S., van der Grond, J., Lechner, A., et al. (2016). Combining anatomical, diffusion, and resting state functional magnetic resonance imaging for individual classification of mild and moderate Alzheimer's disease. *NeuroImage: Clinical* 11, 46–51

Schouten, T. M., Koini, M., de Vos, F., Seiler, S., de Rooij, M., Lechner, A., et al. (2017). Individual classification of Alzheimer's disease with diffusion magnetic resonance imaging. *NeuroImage* 152, 476–481

Seiler, S., Schmidt, H., Lechner, A., Benke, T., Sanin, G., Ransmayr, G., et al. (2012). Driving cessation and dementia: results of the prospective registry on dementia in Austria (PRODEM). *PLoS ONE* 7, e52710

Smith, S. M., Jenkinson, M., Johansen-Berg, H., Rueckert, D., Nichols, T. E., Mackay, C. E., et al. (2006). Tract-based spatial statistics: voxelwise analysis of multi-subject diffusion data. *NeuroImage* 31, 1487–1505

Smith, S. M., Jenkinson, M., Woolrich, M. W., Beckmann, C. F., Behrens, T. E., Johansen-Berg, H., et al. (2004). Advances in functional and structural mr image analysis and implementation as fsl. *NeuroImage* 23, S208–S219

Smith, S. M., Miller, K. L., Moeller, S., Xu, J., Auerbach, E. J., Woolrich, M. W., et al. (2012). Temporally-independent functional modes of spontaneous brain activity. *Proceedings of the National Academy of Sciences* 109, 3131–3136

Song, X.-W., Dong, Z.-Y., Long, X.-Y., Li, S.-F., Zuo, X.-N., Zhu, C.-Z., et al. (2011). Rest: a toolkit for resting-state functional magnetic resonance imaging data processing. *PloS one* 6, e25031

Wink, A. M., de Munck, J. C., van der Werf, Y. D., van den Heuvel, O. A., and Barkhof, F. (2012). Fast eigenvector centrality mapping of voxel-wise connectivity in functional magnetic resonance imaging: implementation, validation, and interpretation. *Brain connectivity* 2, 265–274

Yu-Feng, Z., Yong, H., Chao-Zhe, Z., Qing-Jiu, C., Man-Qiu, S., Meng, L., et al. (2007). Altered baseline brain activity in children with adhd revealed by resting-state functional mri. *Brain and Development* 29,

83–91. doi:https://doi.org/10.1016/j.braindev.2006.07.002

Zhang, Y., Brady, M., and Smith, S. (2001). Segmentation of brain mr images through a hidden markov random field model and the expectation-maximization algorithm. *IEEE transactions on medical imaging* 20, 45–57

Zou, Q.-H., Zhu, C.-Z., Yang, Y., Zuo, X.-N., Long, X.-Y., Cao, Q.-J., et al. (2008). An improved approach to detection of amplitude of low-frequency fluctuation (alff) for resting-state fmri: fractional alff. *Journal of neuroscience methods* 172, 137–141
